# The histo-blood group antigens of the host cell may determine the binding of different viruses such as SARS-CoV-2

**DOI:** 10.2217/fmb-2020-0158

**Published:** 2021-01-18

**Authors:** Mayra Cuéllar-Cruz

**Affiliations:** 1 Departamento de Biología, División de Ciencias Naturales y Exactas, Campus Guanajuato, Universidad de Guanajuato, Noria Alta S/N, Col. Noria Alta, C.P. 36050, Guanajuato, Guanajuato, México

**Keywords:** coronavirus, HBGAs, SARS-CoV-2

## Abstract

Viruses have caused the death of millions of people worldwide. Specifically, human viruses are grouped into 21 families, including the family of coronaviruses (CoVs). In December 2019, in Wuhan, China, a new human CoV was identified, SARS-CoV-2. The first step of the infection mechanism of the SARS-CoV-2 in the human host is adhesion, which occurs through the S glycoprotein that is found in diverse human organs. Another way through which SARS-CoV-2 could possibly attach to the host’s cells is by means of the histo-blood group antigens. In this work, we have reviewed the mechanisms by which some viruses bind to the histo-blood group antigens, which could be related to the susceptibility of the individual and are dependent on the histo-blood group.

Along history, microorganisms have had a massive effect on human activities, mostly in a negative way, because diseases, since ancient times until our present days, control human events, as epidemics cause social, economic and political chaos as well as human suffering. With the development of microbiology as a science, it has been possible to identify microorganisms, develop vaccines, drugs and antiseptic techniques that have allowed controlling most of the known infectious diseases in humans [1–3]. However, microorganisms have an intrinsic plasticity of their genomes to acquire new properties that are, eventually, noxious to humans, in this way they are able to adapt and persist in the diverse physiological niches [4,5]. Some microorganisms in the history of mankind have caused epidemics associated to high mortality rates, for example, the bacterium *Yersinia pestis* ravaged Europe during the Middle Ages, killing millions of people [6]. In 1918–1920, the virus of the A influenza subtype H1N1 (also known as the Spanish flu) caused the death of 50–100 millions of people worldwide [7–9]. Interestingly, it has been reported that viruses have been circulating for thousands of years, as for example the herpes virus that has infected millions of humans [10,11]. Regarding smallpox, its causative virus is one of the most ancient viruses capable of infecting humans, its description goes back to the year 430 BC [12]. In fact, this disease is considered as the most lethal and devastating of history [13]. Notwithstanding, it was not until 1930, with the invention of the electronic microscope, when the virus could be visualized and identified, which drove the study and understanding of the infection mechanisms in diverse hosts [14–16]. The 20th century witnessed the discovery of many diseases caused by viruses and their classification. According to the infection mechanism in the host by the viruses, they are defined as obligated intracellular small parasites, formed by a DNA or RNA genome, enveloped by a protein cover encoded by the virus. Viruses have evolved to reproduce inside the infected cell, because by themselves they are not capable of doing so because they lack the necessary molecular machinery [17]. Viruses are classified according to their morphology, chemical composition and replication mechanisms. Practically, no virus-free species exists; specifically, human viruses are grouped in 21 families [17]. Of the total viruses, 70% correspond to RNA viruses, which usually present high mutation percentages as compared with DNA viruses. DNA viruses are classified in: *Circovirus*, *Parvovirus*, *Hepadnavirus*, *Papovavirus*, *Adenovirus*, *Herpesvirus*, *Poxvirus*. Whereas RNA viruses are classified in: Picornavirus, Astrovirus, Calicivirus, Flavirus, Togavirus, Retrovirus, Reovirus, Bunyavirus, Orthomyxovirus, Arenavirus, Filovirus, Rhabdovirus, Paramyxovirus and Coronavirus families. Although it has been reported that all genera that comprise these families cause infections, we will focus this review on the family of Coronavirus, as these are the causative agents of respiratory and intestinal infections in human and animals with high mortality rates [18,19].

## Coronaviruses

Coronaviruses (CoVs) were identified as pathogens in humans in the mid-sixties, when isolated for the first time from samples of the respiratory tract of adults with a common cold. These viruses constitute the subfamily Orthoviridae, in the family Coronaviridae, order Nidovirales and owe their name to their circular structure from which spikes emerge reminiscent of the solar corona [20–22]. They are enveloped viruses with a positive-sense ssRNA genome with a length of 26–32 kilobases [21]. Its capsid is formed by a lipid bilayer that anchors the structural S proteins (spikes), responsible for the corona shape and for recognizing of receptors in the host’s cell, protein M, which is a membrane glycoprotein; and protein E, which is a small protein implicated in several processes of the viral cycle [23,24].

**Figure 1.**
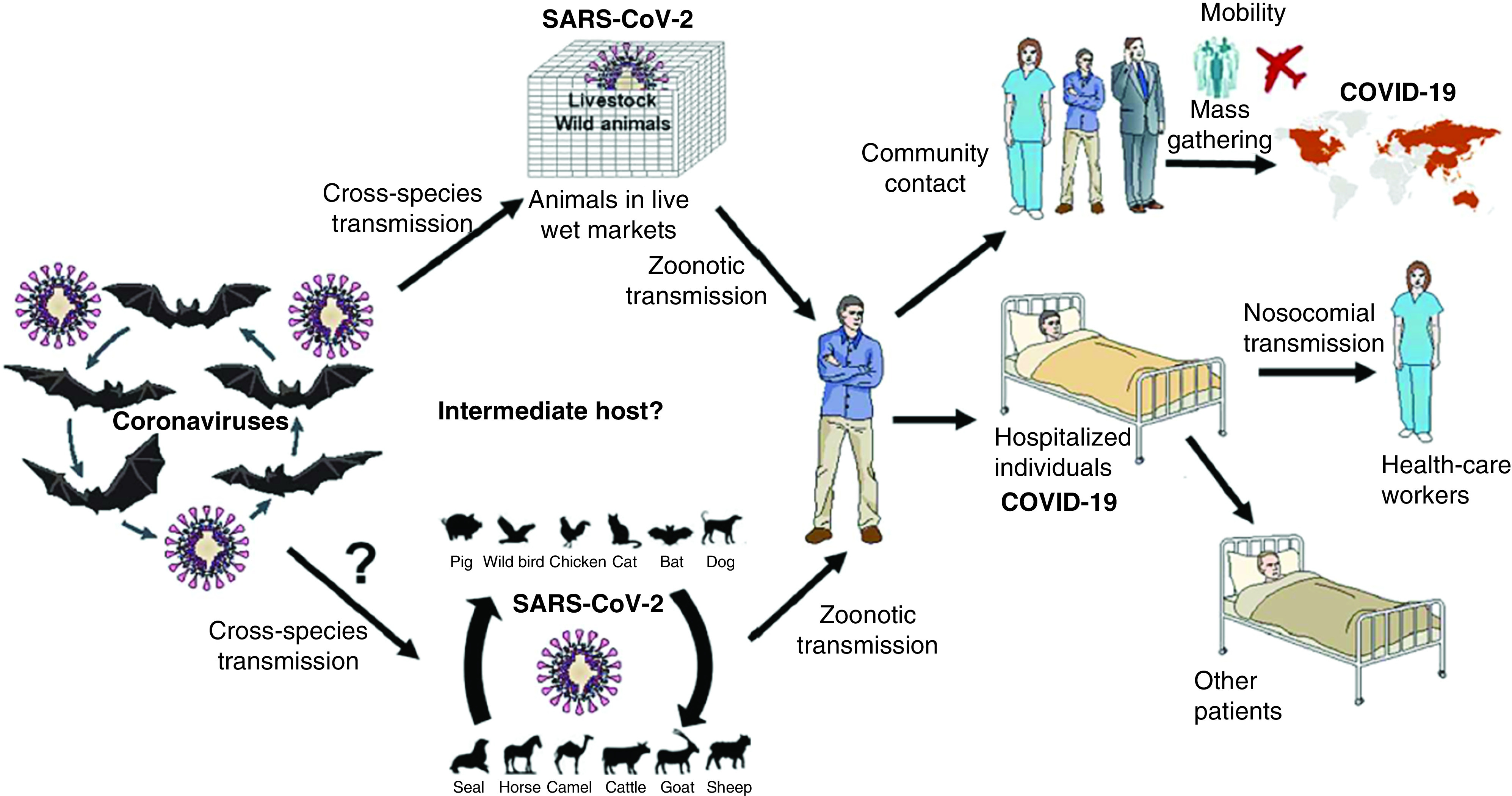
Primary hosts of human coronaviruses. Figure reprinted, with permission, from El Zowalaty and Järhult (2020) [33].

CoVs are currently classified in five genera: *Alphacoronavirus*, *Betacoronavirus*, *Gammacoronavirus*, *Deltacoronavirus* and *Hibecovirus;* they have been subclassified into lineages A, B, C and D [25,26]. These genera have a certain specificity for different hosts. *Alphacoronaviruses* and *Betacoronaviruses* infect only mammals, whereas *Gammacoronaviruses* and *Deltacoronaviruses* infect avians and some can infect mammals [27]. Some of these CoVs are: HCoV-NL63, HCoV-229E, HCoV-OC43, HCoV-HKU1, SARS-CoV, MERS-CoV and SARS-CoV2 [18,28]. CoVs HCoV-229E, HCoV-OC43, HCoV-NL63 and HCoV-HKU1 are present in the common cold together with other pathogens like the rhinoviruses; it is estimated that a high percentage of the population has developed antibodies to them. The fifth type of coronavirus to appear in humans was the SARS-CoV that causes the acute respiratory syndrome. It was identified for the first time in the Guangdong Province, China, in November 2002; in February 2003, the virus spread rapidly from Southern China to Hong Kong and the rest of the world [29–31], it infected approximately 8000 people with a 10% mortality [32]. The sixth type of CoV to appear in 2012 was the one causing the Middle Eastern respiratory syndrome, and called MERS-CoV. This virus shares about 80% of its genome with that of SARS-CoV with a lethality of 34.5%. The seventh type is the SARS-CoV-2, which appeared in December 2019 in Wuhan, China and is currently under research [28]. CoVs have evolved to be able to infect humans, since their natural hosts are wild animals [18], but in the transmission chain to their final host, humans, other animals participate as intermediates (Figure 1). For example, the MERS-CoV had dromedaries as intermediates, between bats and humans [33–35], this was also observed in the epidemics caused by SARS-CoV, in which, at the beginning, patients had contact with animals; antibodies were found in masked palm civets (*Paguma larvata*) and in people that handled these animals in public markets [30,36,37]. Later on, it was found that bats are the natural host of this CoV [26]. Since then until this date, it has been found that bats are the hosts for at least 30 CoVs [34,38,39]. Several events and incidents are involved in viruses switching hosts, like from bats to other animals or humans. For example, one of these events was given by bats being consumed as food in southern China and other countries in Southeast Asia, which is pointed out by the fact that the first reported case of SARS, several years ago, occurred in a chef from Heyuan [26,40]. Recently, the fact that Wuhan, China, was the site of the first case of the new CoV in humans named SARV-CoV-2 [41,42] is an additional indication that this type of event represents a possibility, among others, that the β-CoV be transmitted from the bat to the human host. The transmission from one species to another can be the result of several events (Figure 1) [33].

Although the spillover of CoVs from a wild animal to humans can be explained by the handling and consumption of bats or other wild animals, the entry mechanism that the virus has to exert from one cell to the other is of great interest, as it allows understanding which are the proteins and biomolecules implicated in the process. The first step that CoV must perform, like any other pathogen, to be able to infect the host's cells is adhesion. This first step is performed through the glycoprotein S [23]. Protein S divides into two domains known as S1 and S2. Domain S1 contains a receptor binding domain to the host [43]. The ACE2 is a receptor for SARS-CoV [44]. After binding to the ACE2, the conformational change of protein S induces exposure of domain S2, and in this way helps the virus to fuse with cells of the host [45]. For this reason, the receptor binding domain of domain S1 is considered the main target for the design of peptides and antibodies to neutralize infections by this virus [46]. Because SARS-CoV and SARS-CoV-2 adhere to ACE2 in the host’s cells, these viruses are able to colonize the heart, kidney, lungs, intestine, mouth, esophagus and the gastrointestinal tracts as these organs express this protein. The SARS-CoV-2 virus invades mainly the alveolar epithelial cells, specifically the ACE2 receptors of the alveolar pneumocytes. An *in silico* analysis proposed that the binding of SARS-CoV-2 to ACE2 occurs through the binding of the S1 N terminal domain (S1-NTD) of the spike protein to the glycans that contain sialic acid [47]. Despite that the mechanism by which SARS-CoV adheres to the cells of the human host to infect the diverse organs of the patient is known, in other words, by entering the blood stream and from there to the organs. The precise mechanism by which SARS-CoV adheres to the red blood cells that lack the ACE2 could possibly occur by adhesion to the CD147 receptor, as it has been described that SARS-CoV can bind to the CD147 receptor on the cellular surface [48], which is found in red blood cells. CD147 is a protein of the immunoglobulins, which exert functions like starting the work of metalloproteinases that reconstruct the extracellular substance in tissues. This receptor, aside of being located in the surface of red blood cells, is also found in epithelial, endothelial and leucocyte cells. These data show that the SARS-CoV possesses mechanisms that allow them to colonize efficiently the human cells.

## Correlation between blood type & infection by COVs

Several works have described that the blood type or the ethnicity of an individual plays a role in being more or less susceptible to the infection by this CoV type, as occurs in other diseases. For example, in candidemia, which is caused by opportunistic pathogen fungi of the *Candida* genus, people of African descent, children less than a year old and patients older than 65 all have a higher prevalence of the disease [49–51]. Regarding the participation of the blood type of the individual, it has been described that it influences some diseases either infectious or noninfectious. In this section, we will focus on the blood type and its possible relation with susceptibility to SARS-CoV-2. The ABO was discovered in 1900 by Karl Landsteiner [52] and it is one of the most important systems for transfusions. This system is formed by antigens A, B and their corresponding antibodies, giving rise to four blood groups (A, B, AB and O), in other words, the ABO blood groups, also known as the histo-blood group system. Thanks to the discovery of the ABO system, safe transfusion of blood has been favored and the system has also been used as one of the human hereditary characteristics most important discoveries in medicine, as well as for tests to confirm paternity, in forensic studies, as well as in studies of diverse populations. The distribution of the four blood groups varies in the different populations of the world, group O is the most frequent, followed by group A, group B and group AB [53–55]. Antigens of the ABO system are formed by sugars that are found on the surface of erythrocytes [56,57], bound to the ceramide located in the membrane of erythrocytes. The terminal sugars of each blood group and the antibody they produce are summarized in Table 1.

**Table 1.** Characteristics of the ABO system.

Blood group	Terminal sugars	Antibodies in plasma	Antigens in red blood cells
A	Acetylgalactosamine + fucose	Anti-B	A antigen
B	Galactose + fucose	Anti-A	B antigen
O	Fucose	Anti-A and anti-B	None
AB	Acetylgalactosamine + fucose; galactose + fucose	None	A and B antigens

**Figure 2.**
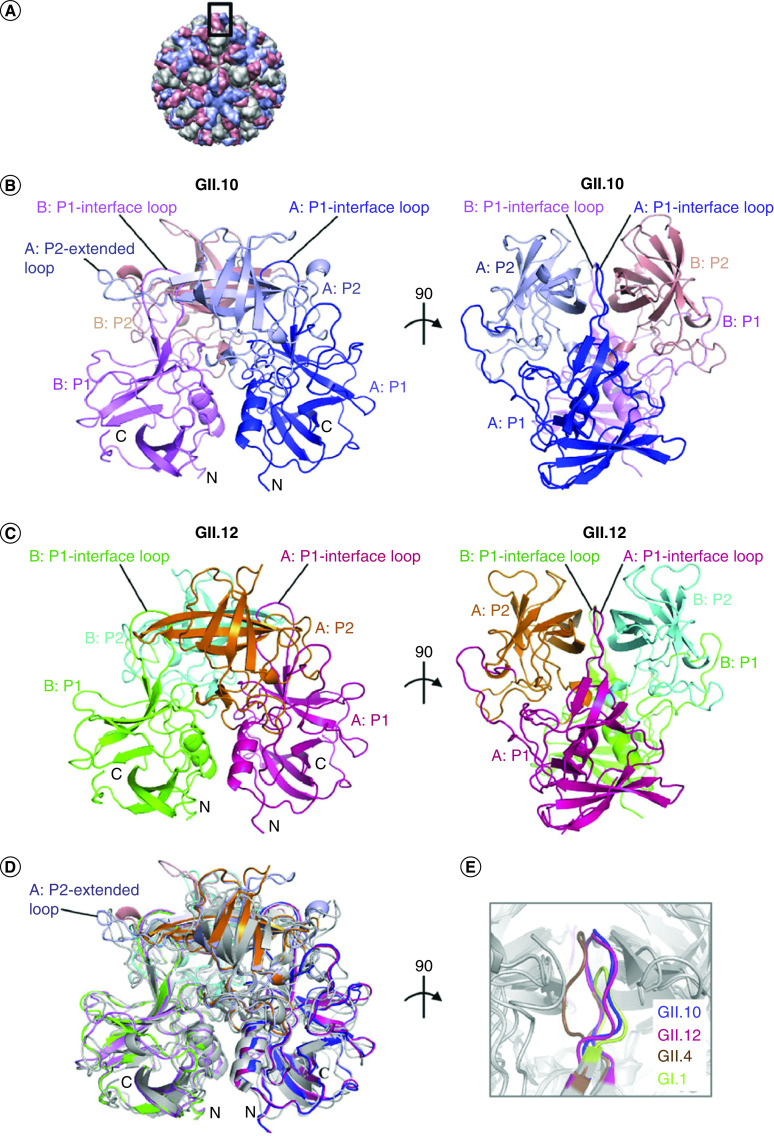
Structures of the norovirus GII.10 and GII.12 P domains. The GII.10 and GII.12 P1 subdomains are very similar, with greater differences observed in the P2 subdomains. **(A)** The GII.10 VLP was modeled from the shell domain of the NV VLP (PDB ID, 1IHM) and the unbound GII.10 P domain (PDB ID, 3ONU). The GII.10 VLP (T3) was modeled with different monomer interactions, A/B and C/C, where each A, B and C monomer was colored light blue, salmon and orange, respectively. The boxed region showed the location of the P domain capsid dimer. **(B)** The x-ray crystal structure of the unbound GII.10 P domain dimer was determined to have 1.4-Å resolution and colored according to monomers (chains A and B) and P1 and P2 subdomains, in other words, chain A P1 (blue), chain A P2 (light blue), chain B P1 (violet) and chain B P2 (salmon). The chain A P2-extended loop protruded out from the side of the P domain (the chain B extended loop was not fitted into the structure). The P1-interface loop was at the dimer interface and surface exposed. **(C)** The x-ray crystal structure of the unbound GII.12 P domain monomer determined to 1.6-Å resolution (shown here as a modeled dimer) was colored according to monomers and P1 and P2 subdomains, in other words, chain AP1 (hot pink), chain A P2 (orange), chain B P1 (green) and chain B P2 (cyan). As for the GII.10 P1-interface loop, the GII.12 P1-interface loop was at the dimer interface and surface exposed. **(D)** Superposition of GII.10 (PDB ID, 3ONU), GII.12 (PDB ID, 3R6J), GII.4 (PDB ID, 2OBR) and GI.1 (PDB ID, 2ZL5), colored as shown in panels B and C, dark gray and light gray, respectively, indicated that the four structures were very similar, except for several differences, including the P1-interface loop and the P2-extended loop. **(E)** The P1-interface loop (a close-up 90° rotation of panel D) was located at a dimer interface for all four structures. The lengths of the P1-interface loops were the same for GII.10, GII.12 and GII.4 but shorter for GI.1 (residues 445 to 456, 432 to 443, 436 to 447 and 425 to 431, respectively). GII: Genotype; NV: Norovirus. Figure and legend reprinted, with permission, from Hansman *et al.* (2011) [70].

Antigens of the ABO system have been associated with susceptibility to different diseases and infections; for example, people with blood group A have a greater susceptibility to develop salivary glands carcinoma, stomach, colon, rectum, ovary, uterus, cervix, bladder carcinomas, idiopathic thrombocytopenic purpura, coronary thrombosis, oral contraceptives-induced thrombosis, giardiasis, meningitis due to meningococcus [58–60]. Whereas individuals of blood group O present more susceptibility to gastric and duodenal ulcers, rheumatoid arthritis, von Willebrand disease, typhoid and paratyphoid disease, cholera and gastrointestinal infections caused by *Escherichia coli* 0157 [58,61–63]. Individuals of blood type B are more susceptible to urinary infections due to *E. coli* and blenorragia [58]. For virus-induced infections, it has been reported that viruses of the Caliciviridae family, which are positive-sense RNA viruses comprised in nine genera, *Vesivirus*, *Lagovirus*, *Norovirus*, *Sapovirus, Nebovirus*, *Recovirus*, *Valovirus*, *Nacovirus* and *Bavovirus* [64,65] are found in several hosts [66–69]. Viruses of the *Norovirus* genus are capable of infecting a wide number of hosts, including humans, canine, feline, ovine, swine, bovine and murine [64,66–69]. Human noroviruses (HuNVs) are responsible for viral gastroenteritis and acute gastroenteritis outbreaks worldwide [65,70]. Interestingly, HuNVs bind to the carbohydrates of the antigens of histo-blood group antigens (HBGAs) before their internalization; besides, it has been reported the polymorphism of the histo-blood antigens is related to the susceptibility presented by the individual to HuNVs [65], pointing out the relevance of the HBGAs in the entry mechanism. Noroviruses have been divided in six different geno-groups based on the sequences of their capsids. HuNVs are comprised in geno-groups I (GI), II and IV [64,65,70]. Although HuNVs, GI and GII, bind to the HBGAs, to elucidate the interaction mechanisms between HuNVs and the HBGAs, the crystalline structures of the HuNV protruding domains of two rarely identified genotypes were determined, these were the Vietnam026 (026) GII.10 and Hiro G11.12 [70]. The crystalline structures were resolved alone and in a complex with a panel of HBGAs (Figure 2).

**Figure 3.**
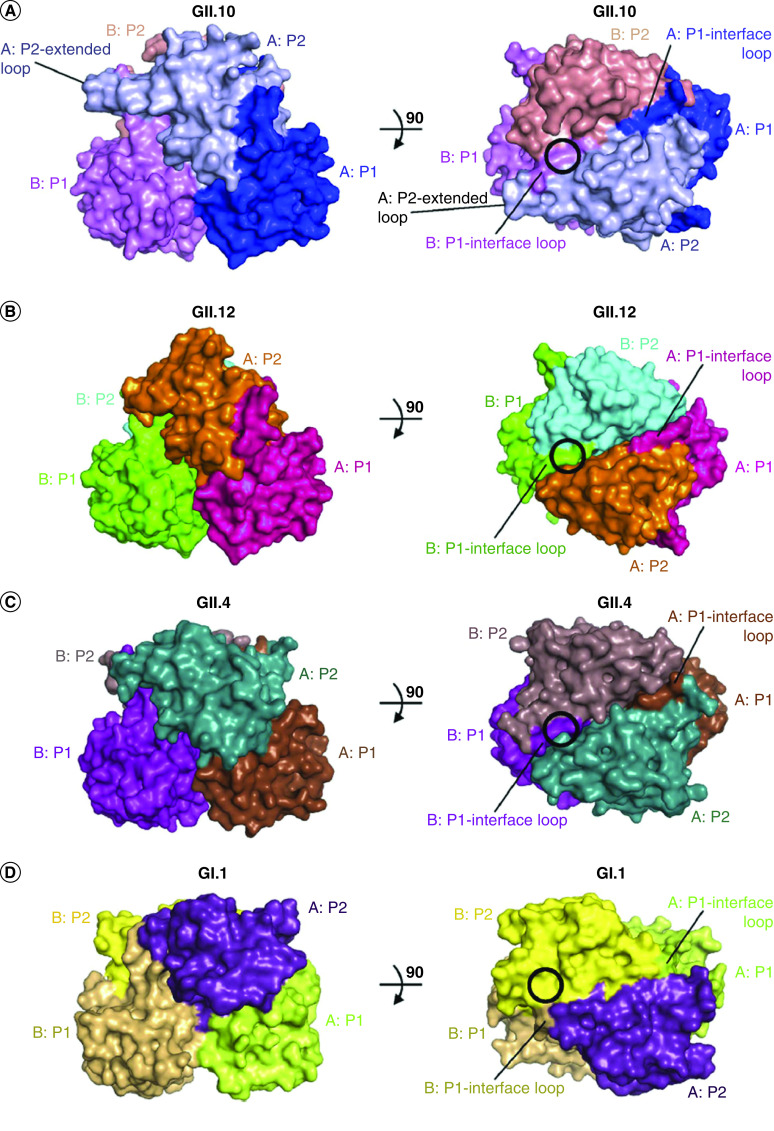
Surface comparisons of the GII.10 (PDB ID, 3ONU), GII.12, Genotype 4 (PDB ID, 2OBR) and GII.1 (PDB ID, 2ZL5) P domain dimer structures. The GII HBGA binding sites (black circles in panels A to C) involve a dimeric capsid interface that is formed primarily by the P2 subdomain and includes a P1-interface loop, whereas the GI HBGA binding site (black circle in panel D) is monomeric, involves only a single P2 subdomain and makes no contact with the P1 subdomain. **(A)** The GII.10 P2 subdomain had an amino acid insertion (relative to those of the other GII sequences), which corresponded to a P2-extended loop. **(B)** The GII.12 P2 subdomain was somewhat unlike the other two GII surfaces, having a more pointed P2 subdomain. **(C)** The GII.4 P2 subdomain was more similar to that of GII.10 but had a less pointed P2 subdomain top surface. **(D)** The GI.1 P domain appears somewhat flatter than that of the GII structures. GII: Genotype; HBGA: Histo-blood group antigen. Figure and legend reprinted, with permission, from Hansman *et al.* (2011) [70].

Results from analyzing different crystalline structures revealed that in the interaction of the norovirus GII with the HBGAs participate six hydrogen bonds between the α-1,2-fucose terminal of the HBGAs and a dimeric capsid interface, which is composed of elements of two protruding subdomains (Figure 3) [70]. The interactions of noroviruses with other saccharide units of the HBGAs involve few hydrogen bonds. In norovirus GII, a conserved residue was identified with α-1,2-fucose, on the external surface of the virion capsid [70].

Another study elucidated the crystalline structure of another HuNV genotype, specifically the geno-group II, genotype 4 (GII.4) [71]. These authors mention that noroviruses evolve every 2 years and it is considered that they modify the binding interaction with different HBGA types. The crystalline structures of the capsid protruding (P) domains of the epidemic variants of years 2004, 2006 and 2012 were studied, which were co-crystallized with a panel of HBGAs to elucidate the binding mechanisms of these viruses with the HBGAs [71]. Domain P is important because it contains the determinants for the binding cells and the diversity of strains. This domain is subdivided in subdomains P1 and P2 and each of them is considered to have unique functions. Binding of P domains of noroviruses G11.4 of 2004, 2006 and 2012 to the HBGAs were analyzed through x-ray crystallography. Results showed that the amino acids variations were localized in subdomain P2 [71]. It was also found that GII.4 viruses are able to bind to different types of HBGAs, this binding is achieved by complex interactions, such as movements of the coil of domain P with the consequent alternative conformations of the HBGAs; for example, a coil (residues 391–395) repositioned to allow for the Lewis Y binding. This same coil displaced itself slightly to provide direct links mediated by Lewis B hydrogen and water. Based on these data, the authors conclude that the outbreaks caused by noroviruses GII.4 are due to highly regulated binding mechanisms to the HBGAs, their affinity for the Lewis-type HBGAs, and their amino acids modifications.

**Figure 4.**
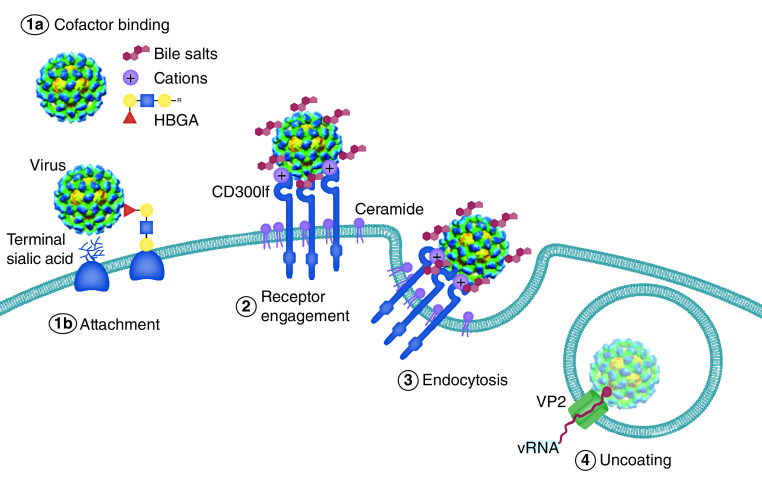
Model of norovirus entry. The first and often rate-limiting step of viral entry is viral attachment to the cell surface. Cell-associated host glycans including terminal sialic acid and HBGAs can facilitate the entry of mouse (MNoV) and HNoV, respectively. Soluble cofactors including soluble forms of HBGAs (HNoV), bile salts (MNoV and HNoV) and divalent cations (MNoV) can also augment the attachment of the virus to cells. For MNoV, these soluble cofactors increase virus attachment in a receptor-dependent manner. The second stage of viral entry is receptor engagement. CD300lf, an Ig domain-containing membrane protein, is the MNoV receptor and fJAM-A is the FCV receptor, while the HNoV receptor remains unknown. Interestingly, ceramide alters CD300lf conformation or clustering, promoting the interaction with MNoV. Following receptor engagement, the virus is endocytosed where, at least for FCV, receptor binding triggers the minor capsid protein VP2 to form a membrane portal that may enable viral genome release in the cytosol. FCV: Feline calicivirus; fJAM-A: Feline junctional adhesion molecule A; HBGA: Histo-blood group antigen; HNoV: Human norovirus. Figure and legend reprinted, with permission, from Graziano *et al.* (2019) [73].

Once the virus has bound to its cellular target, a series of events are released that allow the virus to enter the cell. In general, the steps followed by the virus are: binding to the cellular target; compromise the host; endocytosis; penetration of the cellular membrane; delivery of the viral genome to the cytoplasm [72,73]. Once the virus has been successful in entering the cells, cellular tropism and pathogenesis take place [73]. In this mechanism, one of the key points, perhaps the most relevant, is precisely the binding of the virus to the HBGAs. The molecular mechanism by which these molecules promote infection is proposed to occur as described in Figure 4 [73].

Another type of identified norovirus is the canine norovirus, which has been classified in genogroups IV (GIV) and VI (GVI). A study determined the binding specificity of these noroviruses to carbohydrates; interestingly, α-1,2-fucose was identified as a key factor for the attachment. Through ELISA and immunohistochemistry it was confirmed that the canine norovirus recognizes HBGAs groups that contain α-1,2-fucose [65]. These data support the idea on the evolution of noroviruses, and this could be the key step in the zoonotic transmission from animals to humans.

Another analyzed virus is the BtCalV/A10/USA/2009, which likely represents a novel genus of Caliciviridae. Carbohydrate binding studies were performed with this norovirus and it was found, as with bat noroviruses, that their capsid sequences resembled those of HuNVs, as they retained the binding to the host’s ligand within binding domains to the receptor similarly to HuNVs. This calicivirus binds to the HBGAs, as observed with human and animal noroviruses. These data indicate the ability of bat caliciviruses to bind to HBGAs and, thereby, overcome the significant barrier for transmission among species [64]. Another virus from the Caliciviridae family is the bovine nebovirus, causative agent of acute gastroenteritis in cattle. This virus binds to the HBGAs specifically to those of the type A/H type 2/Le^y^ expressed in the bovine digestive tract; interestingly, they are related to the HBGAs expressed in humans and in other host species [74]. Results also showed that the bovine nebovirus binds to α-1,2-fucose, indicating that this virus can be transmitted to other hosts like humans.

Regarding the binding mechanisms of SARS-CoV-2 to the different HBGAs, it has been shown that depending on the antibodies of the blood groups, these can or not block the interaction between the SARS-CoV-S protein and ACE2 [75]. In this way, it has been shown in experimental models that the binding between ACE2 and SAR-CoV S protein can be blocked specifically by anti-A blood group antibodies, when the S protein was synthetized by cells that express the A histo-blood group antigen [75,76]. These data suggest that for the people with the O blood type, by having anti-A and anti-B antibodies, it could be possible to avoid the infection by blocking the binding and entrance of the virus [75,76]. This observation agrees with a study performed in 2173 patients with COVID-19, which found that individuals with blood type A exhibit a higher susceptibility to become infected with SAR-CoV-2, in contrast to those with the O blood type, which seem to be least susceptible [77].

## Conclusion

Data obtained in this type of studies suggest that this could be one of the binding mechanisms of HBGAs to different types of viruses, like CoVs, SARS-CoV and SARS-CoV-2, which could be related to the susceptibility of the individual, dependent on the histo-blood group.

## Future perspective

HBGAs are important for the adhesion of viruses to the host's cells, as this is the key point to be able to enter the cell and cause the infection. Additionally, binding of the virus to HBGAs is possibly the mechanism used by the virus to go from one host to another, for example, from bats to humans or from bats to an intermediate animal and, finally, to humans. For SARS-CoV and SARS-CoV-2 viruses, formal studies have been started to ascertain whether the binding of these CoVs to HBGAs favors or not the susceptibility of each individual to these CoVs and whether it is an alternative pathway for the binding of SARS-CoV-2 to human cells. This knowledge will allow for the development of a potential vaccine, as well as for the design of new drugs for the treatment of this novel CoV.

**Executive summary**

**Introduction**

- Microorganisms are the cause of pandemics that have affected mankind along its history.
- Viruses have caused the death of millions of people worldwide, as happened with the influenza A of the H1N1 subtype during 1918–1920.
- The 20th century witnessed the discovery of many diseases caused by viruses and their classification.
- Viruses have evolved to reproduce inside the infected cell, because by themselves they are not capable of doing so because they lack the necessary molecular machinery.

**Coronaviruses**

- Viruses are classified according to their morphology, chemical composition and replication mechanisms.
- Specifically, human viruses are grouped into 21 families, among them is the family of coronaviruses (CoVs).
- These viruses were first identified in the 70s and are responsible for respiratory and intestinal infections in humans and animals with high mortality rates.
- One of the CoV types that appeared in humans was the SARS-CoV responsible for the acute respiratory distress, which infected approximately 8000 people.
- Recently, in December 2019, in Wuhan, China, a new human CoV was identified, called SARS-CoV-2, which has a mortality of approximately 2–3%.
- Several events and incidents are involved in viruses switching hosts, like from bats to other animals or humans.
- The first step of the infection mechanism of the SARS-CoV-2 in the human host is adhesion, which occurs through the S glycoprotein that is found in diverse human organs.

**Correlation between blood type & infection by COVs**

- Another way through which SARS-CoV-2 could possibly attach to the host's cells is by means of the histo-blood group antigens (HBGAs).
- The ABO was discovered in 1900 by Karl Landsteiner.
- The distribution of the four blood groups varies in the different populations of the world.
- Antigens of the ABO system have been associated with susceptibility to different diseases and infections.
- For virus-induced infections, it has been reported that viruses of the Caliciviridae family, which are positive-sense RNA viruses comprised in nine genera, *Vesivirus*, *Lagovirus*, *Norovirus*, *Sapovirus, Nebovirus*, *Recovirus*, *Valovirus*, *Nacovirus* and *Bavovirus* are found in several hosts.
- Viruses of the *Norovirus* genus are capable of infecting a wide number of hosts, including humans, canine, feline, ovine, swine, bovine and murine.
- Human noroviruses (HuNVs) are responsible for viral gastroenteritis and acute gastroenteritis outbreaks worldwide.
- Interestingly, HuNVs bind to the carbohydrates of the antigens of HBGAs before their internalization.
- Noroviruses have been divided in six different geno-groups based on the sequences of their capsids. HuNVs are comprised in geno-groups I (GI), II, and IV.
- Results from analyzing different crystalline structures revealed that in the interaction of the norovirus GII with the HBGAs participate six hydrogen bonds between the α-1,2-fucose terminal of the HBGAs and a dimeric capsid interface, which is composed of elements of two protruding subdomains.

**Future perspective**

- HBGAs are important for the adhesion of viruses to the host’s cells, as this is the key point to be able to enter the cell and cause the infection.
- Besides, binding of the virus to HBGAs is possibly the mechanism used by the virus to go from one host to another, for example, from bats to humans.
- For SARS-CoV and SARS-CoV-2 viruses, it has been formally studied whether the binding of these coronaviruses to HBGAs favors or not the susceptibility of each individual to these CoVs.

## References

[1] Mäkelä PH Vaccines, coming of age after 200 years. FEMS Microbiol. Rev. 24(1), 9–20 (2000). 10640596 10.1111/j.1574-6976.2000.tb00530.x

[2] Oyston P, Robinson K The current challenges for vaccine development. J. Med. Microbiol. 61(Pt 7), 889–894 (2012). 22322337 10.1099/jmm.0.039180-0

[3] Guthertz LS Teaching the history of microbiology and the transformation of the laboratory: a study in miniature. J. Microbiol. Biol. Educ. 18(1), 1–7 (2017). 10.1128/jmbe.v18i1.1266 PMC5524440 28904644

[4] Cui Y, Song Y Genome and evolution of *Yersinia pestis*. Adv. Exp. Med. Biol. 918, 171–192 (2016). 27722863 10.1007/978-94-024-0890-4_6

[5] Achtman M, Morelli G, Zhu P Microevolution and history of the plague bacillus, *Yersinia pestis*. Proc. Natl Acad. Sci. USA 101(51), 17837–17842 (2004). 15598742 10.1073/pnas.0408026101 PMC535704

[6] Titball RW, Hill J, Lawton DG, Brown KA *Yersinia pestis* and plague. Biochem. Soc. Trans. 31(Pt 1), 104–107 (2003). 12546664 10.1042/bst0310104

[7] Morens DM, Taubenberger JK Influenza cataclysm 1918. N. Engl. J. Med. 379(24), 2285–2287 (2018). 30575465 10.1056/NEJMp1814447 PMC6558650

[8] Pederson T Where are we, a century after the “Spanish Flu”?. FASEB J. 32(5), 2317–2318 (2018). 29698119 10.1096/fj.180501ufm

[9] Honigsbaum M Spanish influenza redux: revisiting the mother of all pandemics. Lancet 391(10139), 2492–2495 (2018). 29976462 10.1016/S0140-6736(18)31360-6

[10] Crawford DH The Invisible Enemy: a Natural History of Viruses. Oxford University Press, Oxford, UK (2000).

[11] Crawford DH Viruses: a Very Short Introduction. Oxford University Press, Oxford, UK (2011).

[12] Zimmer C A Planet of Viruses (*2nd Edition*). University of Chicago Press, IL, USA (2011).

[13] Riedel S Edward Jenner and the history of smallpox and vaccination. Proc. (Bayl. Univ. Med. Cent.) 18(1), 21–25 (2005). 16200144 10.1080/08998280.2005.11928028 PMC1200696

[14] Okada Y Tobacco mosaic virus: pioneering research for a century. Tanpakushitsu Kakusan Koso 45(10), 1757–1765 (2000). 10897688

[15] Zhang Z A brief history of discovering virus. Zhonghua Yi Shi Za Zhi 30(2), 90–92 (2000). 11624646

[16] Artenstein AW The discovery of viruses: advancing science and medicine by challenging dogma. Int. J. Infect. Dis. 16(7), e470–e473 (2012). 22608031 10.1016/j.ijid.2012.03.005

[17] Gelderblom HR Structure and classification of viruses, chapter 41. : Medical Microbiology (*4th Edition*). Baron S (). University of Texas Medical Branch at Galveston, Galveston, TX, USA (1996). 21413309

[18] Cui J, Li F, Shi ZL Origin and evolution of pathogenic coronaviruses. Nat. Rev. Microbiol. 17(3), 181–192 (2019). 30531947 10.1038/s41579-018-0118-9 PMC7097006

[19] Masters PS, Perlman S Fields Virology. Knipe DM, Howley PM (). Lippincott Williams & Wilkins, PA, USA, 825–858 (2013).

[20] González JM, Gomez-Puertas P, Cavanagh D, Gorbalenya AE, Enjuanes LA Comparative sequence analysis to revise the current taxonomy of the family *Coronaviridae*. Arch. Virol. 148(11), 2207–2235 (2003). 14579179 10.1007/s00705-003-0162-1 PMC7087110

[21] Lai MM, Holmes C, Holmes KV Coronaviruses. : Fields Virology. Knipe DM, Howley PM, Griffin DE, Lamb RA, Martin MA, Roizman B, Straus SE (). Lippincott Williams & Wilkins, PA, USA, 1163–1185 (2001).

[22] Vijaykrishna D, Smith GJ, Zhang JX, Peiris JS, Chen H, Guan Y Evolutionary insights into the ecology of coronaviruses. J. Virol. 81(8), 4012–4020 (2007). 17267506 10.1128/JVI.02605-06 PMC1866124

[23] Marra MA, Jones SJ, Astell CR The genome sequence of the SARS-associated coronavirus. Science 300(5624), 1399–1404 (2003). 12730501 10.1126/science.1085953

[24] Ruan YJ, Wei CL, Ee AL Comparative full-length genome sequence analysis of 14 SARS coronavirus isolates and common mutations associated with putative origins of infection. Lancet 361(9371), 1779–1785 (2003). 12781537 10.1016/S0140-6736(03)13414-9 PMC7140172

[25] De Groot RJ, Baker SC, Baric R Family Coronaviridae. : Virus Taxonomy, Classification and Nomenclature of Viruses. Ninth Report of the International Committee on Taxonomy of Viruses (*1st Edition*). King AMQ, Adams MJ, Carstens EB, Lefkowitz EJ (). Elsevier Academic Press, CA, USA, 806–828 (2011).

[26] Wong ACP, Li X, Lau SKP, Woo PCY Global epidemiology of bat coronaviruses. Viruses. 11(2), 174 (2019). 10.3390/v11020174 PMC6409556 30791586

[27] Woo PC, Lau SKP, Lam CSF Discovery of seven novel mammalian and avian coronaviruses in the genus deltacoronavirus supports bat coronaviruses as the gene source of alphacoronavirus and betacoronavirus and avian coronaviruses as the gene source of gammacoronavirus and deltacoronavirus. J. Virol. 86(7), 3995–4008 (2012). 22278237 10.1128/JVI.06540-11 PMC3302495

[28] Zhou P, Yang XL, Wang XG A pneumonia outbreak associated with a new coronavirus of probable bat origin. Nature 579, 270–273 (2020). 32015507 10.1038/s41586-020-2012-7 PMC7095418

[29] WHO. Summary of probable SARS cases with onset of illness from 1 November 2002 to 31 July 2003. http://www.who.int/publications/m/item/summary-of-probable-sars-cases-with-onset-of-illness-from-1-november-2002-to-31-july-2003

[30] Guan Y, Zheng BJ, He YQ Isolation and characterization of viruses related to the SARS coronavirus from animals in southern China. Science 302(5643), 276–278 (2003). 12958366 10.1126/science.1087139

[31] Ng OW, Tan YJ Understanding bat SARS-like coronaviruses for the preparation of future coronavirus outbreaks – implications for coronavirus vaccine development. Hum. Vaccin. Immunother. 13(1), 186–189 (2017). 27644155 10.1080/21645515.2016.1228500 PMC5287300

[32] Peiris JS, Guan Y, Yuen KY Severe acute respiratory syndrome. Nat. Med. 10, S88–S97 (2004). 15577937 10.1038/nm1143 PMC7096017

[33] El Zowalaty ME, Jarhult JD From SARS to COVID-19: a previously unknown SARS-related coronavirus (SARS-CoV-2) of pandemic potential infecting humans-Call for a one health approach. One Health 9, 1–6 (2020). 10.1016/j.onehlt.2020.100124 PMC7075990 32195311

[34] Corman VM, Ithete NL, Richards LR Rooting the phylogenetic tree of middle east respiratory syndrome coronavirus by characterization of a conspecific virus from an african bat. J. Virol. 88(19), 11297–11303 (2014). 25031349 10.1128/JVI.01498-14 PMC4178802

[35] Anthony SJ, Gilardi K, Menachery VD Further evidence for bats as the evolutionary source of middle east respiratory syndrome coronavirus. mBio 8(2), e00373–17 (2017). 28377531 10.1128/mBio.00373-17 PMC5380844

[36] Kan B Molecular evolution analysis and geographic investigation of severe acute respiratory syndrome coronavirus-like virus in palm civets at an animal market and on farms. J. Virol. 79(18), 11892–11900 (2005). 16140765 10.1128/JVI.79.18.11892-11900.2005 PMC1212604

[37] Tu C, Crameri G, Kong X Antibodies to SARS coronavirus in civets. Emerg. Infect. Dis. 10(12), 2244–2248 (2004). 15663874 10.3201/eid1012.040520 PMC3323399

[38] Lau SKP, Li KSM, Huang Y Ecoepidemiology and complete genome comparison of different strains of severe acute respiratory syndrome-related rhinolophus bat coronavirus in China reveal bats as a reservoir for acute, self-limiting infection that allows recombination events. J. Virol. 84(6), 2808–2819 (2010). 20071579 10.1128/JVI.02219-09 PMC2826035

[39] Lau SK, Woo PC, Li KS Complete genome sequence of bat coronavirus HKU2 from Chinese horseshoe bats revealed a much smaller spike gene with a different evolutionary lineage from the rest of the genome. Virology 367(2), 428–439 (2007). 17617433 10.1016/j.virol.2007.06.009 PMC7103351

[40] Zhao GP SARS molecular epidemiology: a Chinese fairy tale of controlling an emerging zoonotic disease in the genomics era. Philos. Trans. R. Soc. Lond. B Biol. Sci. 362(1482), 1063–1081 (2007). 17327210 10.1098/rstb.2007.2034 PMC2435571

[41] Velavan TP, Meyer CG The COVID-19 epidemic. Trop. Med. Int. Health 25(3), 278–280 (2020). 32052514 10.1111/tmi.13383 PMC7169770

[42] Guo YR, Cao QD, Hong ZS The origin, transmission and clinical therapies on coronavirus disease 2019 (COVID-19) outbreak – an update on the status. Mil. Med. Res. 7(1), 11 (2020). 32169119 10.1186/s40779-020-00240-0 PMC7068984

[43] Gallagher TM, Buchmeier MJ Coronavirus spike proteins in viral entry and pathogenesis. Virology 279(2), 371–374 (2001). 11162792 10.1006/viro.2000.0757 PMC7133764

[44] Li W, Moore MJ, Vasiliev N Angiotensin-converting enzyme 2 is a functional receptor for the SARS coronavirus. Nature 426(6965), 450–454 (2003). 14647384 10.1038/nature02145 PMC7095016

[45] Matsuyama S, Taguchi F Receptor-induced conformational changes of murine coronavirus spike protein. J. Virol. 76(23), 11819–11826 (2002). 12414924 10.1128/JVI.76.23.11819-11826.2002 PMC136913

[46] Chang HH, Chen PK, Lin GL Cell adhesion as a novel approach to determining the cellular binding motif on the severe acute respiratory syndrome coronavirus spike protein. J. Virol. Methods 201, 1–6 (2014). 24530430 10.1016/j.jviromet.2014.01.022 PMC7113645

[47] Robson B Bioinformatics studies on a function of the SARS-CoV-2 spike glycoprotein as the binding of host sialic acid glycans. Comput. Biol. Med. 122, 103849 (2020). 32658736 10.1016/j.compbiomed.2020.103849 PMC7278709

[48] Chen Z, Mi L, Xu J Function of HAb18G/CD147 in invasion of host cells by severe acute respiratory syndrome coronavirus. J. Infect. Dis. 191(5), 755–760 (2005). 15688292 10.1086/427811 PMC7110046

[49] Hajjeh RA, Sofair AN, Harrison LH Incidence of bloodstream infections due to *Candida* species and *in vitro* susceptibilities of isolates collected from 1998 to 2000 in a population-based active surveillance program. J. Clin. Microbiol. 42(4), 1519–1527 (2004). 15070998 10.1128/JCM.42.4.1519-1527.2004 PMC387610

[50] Kao AS, Brandt ME, Pruitt WR The epidemiology of candidemia in two United States cities: results of a population-based active surveillance. Clin. Infect. Dis. 29(5), 1164–1170 (1999). 10524958 10.1086/313450

[51] Viudes A, Peman J, Canton E, Ubeda P, Lopez-Ribot JL, Gobernado M Candidemia at a tertiary-care hospital: epidemiology, treatment, clinical outcome and risk factors for death. Eur. J. Clin. Microbiol. Infect. Dis. 21(11), 767–774 (2002). 12461585 10.1007/s10096-002-0822-1

[52] Schwarz HP, Dorner F Karl Landsteiner and his major contributions to haematology. Br. J. Haematol. 121(4), 556–565 (2003). 12752096 10.1046/j.1365-2141.2003.04295.x

[53] Garratty G, Glynn SA, McEntire R ABO and Rh(D) phenotype frequencies of different racial/ethnic groups in the United States. Transfusion 44(5), 703–706 (2004). 15104651 10.1111/j.1537-2995.2004.03338.x

[54] Mourant AE, Kopec AC, Sobczak DK The Distribution of the Human Blood Groups and Other Biochemical Polymorphisms (*2nd Edition*). Oxford University Press, Oxford, UK (1976).

[55] Gibbs MB, Akeroyd JH, Zapf JJ Quantative subgroups of the B antigen in man and their occurrence in three racial groups. Nature 192, 1196–1197 (1961). 10.1038/1921196b0 13898361

[56] Hosoi E Biological and clinical aspects of ABO blood group system. J. Med. Invest. 55(3–4), 174–182 (2008). 18797129 10.2152/jmi.55.174

[57] Watkins W Molecular basis of antigenic specificity in the ABO, H and Lewis blood group systems. : Glycoproteins. Montreuil H, Vliegenhart JFG, Schachter H (). Elsevier, Amsterdam, The Netherlands (1995).

[58] Reid ME, Calhoun L, Petz LD Erytrocyte antigens and antibodies. : Williams Hematology (*7th Edition*). Lichtman MA, Beutler E, Kipps TJ, Seligsohn U, Kaushansky K, Prchal JT (). McGraw-Hill, NY, USA (2005).

[59] Tregouet DA, Heath S, Saut N Common susceptibility alleles are unlikely to contribute as strongly as the FV and ABO loci to VTE risk: results from a GWAS approach. Blood 113(21), 5298–5303 (2009). 19278955 10.1182/blood-2008-11-190389

[60] Jenkins PV, O'Donnell JS ABO blood group determines plasma von Willebrand factor levels: a biologic function after all?. Transfusion 46(10), 1836–1844 (2006). 17002642 10.1111/j.1537-2995.2006.00975.x

[61] Blackwell CC, Dundas S, James VS Blood group and susceptibility to disease caused by *Escherichia coli* O157. J. Infect. Dis. 185(3), 393–396 (2002). 11807723 10.1086/338343

[62] Harris JB, Khan AI, LaRocque RC Blood group, immunity, and risk of infection with *Vibrio cholerae* in an area of endemicity. Infect. Immun. 73(11), 7422–7427 (2005). 16239542 10.1128/IAI.73.11.7422-7427.2005 PMC1273892

[63] Kaper JB, Morris JG Jr, Levine MM Cholera. Clin. Microbiol. Rev. 8(1), 48–86 (1995). 7704895 10.1128/cmr.8.1.48 PMC172849

[64] Kocher JF, Lindesmith LC, Debbink K Bat caliciviruses and human noroviruses are antigenically similar and have overlapping histo-blood group antigen binding profiles. mBio 9(3), e00869–18 (2018). 29789360 10.1128/mBio.00869-18 PMC5964351

[65] Caddy S, Breiman A, le Pendu J, Goodfellow I Genogroup IV and VI canine noroviruses interact with histo-blood group antigens. J. Virol. 88(18), 10377–10391 (2014). 25008923 10.1128/JVI.01008-14 PMC4178834

[66] Farkas T, Sestak K, Wei C, Jiang X Characterization of a rhesus monkey calicivirus representing a new genus of *Caliciviridae*. J. Virol. 82(11), 5408–5416 (2008). 18385231 10.1128/JVI.00070-08 PMC2395209

[67] L'Homme Y, Sansregret R, Plante-Fortier E Genomic characterization of swine caliciviruses representing a new genus of *Caliciviridae*. Virus Genes 39(1), 66–75 (2009). 19396587 10.1007/s11262-009-0360-3

[68] Day JM, Ballard LL, Duke MV, Scheffler BE, Zsak L Metagenomic analysis of the turkey gut RNA virus community. Virol. J. 7, 313 (2010). 21073719 10.1186/1743-422X-7-313 PMC2991317

[69] Wolf S, Reetz J, Otto P Genetic characterization of a novel calicivirus from a chicken. Arch. Virol. 156(7), 1143–1150 (2011). 21404111 10.1007/s00705-011-0964-5

[70] Hansman GS, Biertümpfel C, Georgiev I Crystal structures of GII.10 and GII.12 norovirus protruding domains in complex with histo-blood group antigens reveal details for a potential site of vulnerability. J. Virol. 85(13), 6687–6701 (2011). 21525337 10.1128/JVI.00246-11 PMC3126497

[71] Singh BK, Leuthold MM, Hansman GS Human noroviruses’ fondness for histo-blood group antigens. J. Virol. 89(4), 2024–2040 (2015). 25428879 10.1128/JVI.02968-14 PMC4338890

[72] Marsh M, Helenius A Virus entry: open sesame. Cell 124(4), 729–740 (2006). 16497584 10.1016/j.cell.2006.02.007 PMC7112260

[73] Graziano VR, Wei J, Wilen CB Norovirus attachment and entry. Viruses 11(6), 495 (2019). 10.3390/v11060495 PMC6630345 31151248

[74] Cho EH, Soliman M, Alfajaro MM Bovine nebovirus interacts with a wide spectrum of histo-blood group antigens. J. Virol. 92(9), e02160–17 (2018). 29467317 10.1128/JVI.02160-17 PMC5899197

[75] Breiman P, Ruvën-Clouet N, Le Pendu J Harnessing the natural anti-glycan immune response to limit the transmission of enveloped viruses such as SARS-CoV-2. PLoS Pathog. 16(5), e1008556 (2020). 32437478 10.1371/journal.ppat.1008556 PMC7241692

[76] Guillon P, Clément M, Sébille V, Rivain J-G Inhibition of the interaction between the SARS-CoV spike protein and its cellular receptor by anti-histo-blood group antibodies. Glycobiology 18(12), 1085–1093 (2008). 18818423 10.1093/glycob/cwn093 PMC7108609

[77] Zhao J, Yang Y, Huang H-P Relationship between the ABO blood group and the COVID-19 susceptibility. medRxiv (2020) (Epub ahead of print).

